# Observations on the Etiology and Treatment of War Neurosis

**Published:** 1917-11

**Authors:** 


### NERVOUS AND MENTAL

---

***Observations on the Etiology and Treatment of War Neurosis.*** By Arthur F. HURST, Temporary Major R. A. M. C., Neurologist to Guy's Hospital and to the Royal Victoria Hospital, Netley. *British Medical Journal*, Sept. 29, 1917.

The subject is considered under three main heads. The first is *Exhaustion Resulting in Neurasthenia and Soldier's Heart.* The author notes that since the battle of the Marne there has been comparatively little nervous exhaustion from sheer physical fatigue, except in the Gallipoli and Mesopotamia campaigns. Good sanitation in France has greatly reduced acute and chronic infections, which are a cause of nervous exhaustion; but he notes that comparatively slight toxemia from diarrhea, trench fever, or influenza, may produce exhaustion, which might easily be avoided by timely rest. The most serious causes are mental strain, severe pain, and acute infections, because of their direct action upon the nervous system, and indirectly by their action upon the suprarenal glands, the liver, and probably the thyroid. Continued muscular exertion, great fear and anger, or any great emotion, may lead to chromatolysis of the brain cells. *Symptoms* are the usual ones of civil life. Those described under the name of "soldier's heart" are prominent. Generally the blood pressure is low. *Treatment* should at first consist in complete mental and physical rest in bed until the patient no longer feels tired (generally from one to three weeks, although badly complicated cases may require as many months). A dose of bromide gr. 5 maybe administered two or three times daily. Opium and alcohol must never be used. Insomnia should be carefully prevented by some such treatment as sodium diethyl-barbiturate (medinal) combined with acetyl salicylic acid (aspirin) gr. 15, which the author has found best. 10 to 15 grains of the sodium salt should be given the first night, and reduced by 1 gr. every other night until only the aspirin is used. This may then also be reduced. In extreme cases suggestion under hypnosis is helpful. Once the patient is out of bed, the author believes that

> "The cure for this ill is not to sit still
> And frowst with a book by the fire;
> But to take a large hoe, and a shovel also
> And dig till you gently perspire."

II. *Emotions Resulting in Stupor and Amnesia, Psychasthenia, Hysteria, Hyperadrenalism and Hyperthyroidism, and Exaggerated Defensive Reflexes.* Generally such collapses result from cumulative tension of fear and horror rather than from single incidents. Even when a single event is the cause, it is difficult to discover, because at first the patient often refuses to talk of it, and later it is often obliterated from memory.

*Stupor and Amnesia.* Onset may be gradual, shown in sluggishness and silence, and after special horrors may lead to mental confusion so great that the patient becomes wholly oblivious and wanders sometimes for miles. (Such effects are often produced by shell shock, exhaustion, and epileptic attack.) The processes of nature become involuntary. Stupor may last from a few minutes to a week, and may pass away suddenly, or, as is more usual, by slow degrees.

*Psychasthenia.* — Nightmare, day-dreams, and, less frequently, obsessions and phobias are the usual forms of this affliction. Tics are rare. *Treatment* should in most cases be like that for Neurasthenia, except that hypnotic suggestion and "therapeutic conversations" must play a more important part, and are often the only means of preventing dreams and inducing sleep. Especial care should be exercised to gain the patient's confidence. Tics are most difficult to treat and hypnotic suggestion seems of little avail. Reeducation is the only resort. Regular exercises of the affected muscles and voluntary inhibition for definite periods several times a day give good results. Eructation and aerophagy may be cured by clenching a stick between the teeth whenever the yearning is felt.

*Hysterical Symptoms.* — These are generally caused by the autosuggestion of the physical results of extreme fear, especially that in which the knees give way, the breath is held, and speech is impossible. Hysterical paraplegia or mutism results. Usually a period elapses between the causal incident and the onset, and therefore cases are more frequent in clearing stations and base hospitals than in the trenches. Abnormal suggestibility following a prolonged dazed condition tends to make a patient exaggerate and perpetuate any physical weakness or defect: thus immobility often suggests paralysis, and silence caused by weakness often suggests mutism. Hence hysterical phenomena often result in war cases from causes that in ordinary life do not give rise to them; as, for instance, operations for appendicitis, and cases of muscular rheumatism. *Treatment.* — Since this affection, under whatever form it may appear, is due to suggestion, its best cure is suggestion or persuasion. Although many cases recover spontaneously, some, which cannot at first be distinguished from the others, persist indefinitely unless vigorous treatment is applied. This should be instituted *at once.* Although speedy improvement often follows treatment, complete recovery may be very slow. Patient reeducation for several weeks is often necessary, especially when the gait or speech is affected. Physicians should be on their guard against organic causes and malingering. Too great sympathy and luxury in confortable hospitals often invites the feigning of such symptoms; therefore the patient should be given to understand that there is nothing unusual about his case, and that speedy recovery is expected. Encouragement from men in the same ward who are already improved is a form of suggestion of immense importance. Suggestion, then, in whatever form, is the best and surest treatment. Electricity and massage should be used sparingly, or when applied, they should be vigorous enough to produce immediate effects. Five minutes is often enough to cure a paraplegic man, if he is first aided in making movements, and then is told that he is strong enough to make them himself. In cases where recovery is delayed by clonic spasms, anesthesia followed immediately by passive, then active movements of the patient's limbs, has given quick and lasting results. In mute or aphonic cases, first suggest the certainty of recovery, and then apply, if it is thought best, an intralaryngal electrode, previously connected with a faradic battery. The powerful suggestion caused by the pain and muscle contraction will generally produce the desired results. If electricity has already failed, ether should be given so rapidly as to cause great excitement. If the patient does not then talk spontaneously an electrode may be used, and the patient made to talk continuously until the anesthesia has passed. Similar treatment may cure stammering if applied at once, but generally reeducation is needed with lessons in breathing and talking every day. Hysterical fits may be diagnosed from epilepsy, if one is induced by the physician by hypnotic suggestion. The author has found it effective to tell the patient before, during, and after the fit so induced, that it will be the last one that the patient will have. Cases of absolute and genuine hysterical deafness have been cured by pretending to operate on the patient. Enough ether has been given in such cases to make the patient excited, then two small cuts have been made behind the ear, and a hammer banged on a sheet of iron. In one case the patient jumped from the operating table in great joy although he had been deaf for nine months, and had been unable to hear the loudest noises even in sleep. By similar suggestion, blindness has been cured.

*Hyperadrenalism and Hyperthyroidism.* — Such cases result from a failure to expend the tremendous energy which the emotions of anger and fear, especially fear, supply to the muscles through a sympathetic nervous energy which causes abnormal secretions from the suprarenal and thyroid glands. When properly expended, this energy permits comparatively weak bodies to accomplish tremendous feats. They become entirely useless, however, if the emotions are not accompanied by the activity to which they instinctively give rise—that is, anger to fighting, and fear to flight. The author goes on to say: "Thus the ceaseless fear felt by the constitutionally timid when exposed to the horrors of war results in constant over-secretion of the suprarenal and thyroid glands, the physiological results of which are not followed by the muscular activity of flight for which they are the preparation. The unexpected energy may be so extreme that the soldier is incapacitated by it. On reaching the safety of a base hospital the hyperactivity of the suprarenal and thyroid glands and the signs and symptoms to which they give rise often disappear. But they may be perpetuated by war dreams and in severe cases the mind is absorbed by day as well as by night by pictures of the horrors which the individual has witnessed; every sound reminds him of shells and every movement suggests the approach of danger. The activity of the suprarenal and thyroid glands is maintained, and the patient presents a picture suggestive of Graves's disease ". Rapid pulse, enlargement of the heart, and high blood pressure, are attending phenomena.

A pilomotor reflex can be produced sometimes by touching the skin so lightly that under any other conditions no effect would be produced. Goose skin appears and the hair sometimes stands on end. This phenomenon in followed by a vaso-dilator reflex, a blush with white borders. " Circulatory symptoms may be so marked that the case is often diagnosed as ' disordered action of the heart'. Excessive sweating often occurs, sometimes in paroxysms.... The hands and occasionally the eyelids are tremulous, and the patient is highly nervous and excitable. The eyes are often slightly prominent, and von Graefe's sign may be obtained. " *Treatment.* The patient should be isolated from other patients by screens, and he should see only persons likely to allay his irritability. He never should be even remotely reminded of the experiences he has passed through. Opium in this as in no other war neurosis may be used to allay mental and nervous activity. Suggestion is helpful when nightmares or special anxiety are involved. Belladonna tends to reduce the secretions. X-ray applications have been sometimes successful in affecting the thyroid gland, but they are likely to be otherwise injurious.

*Exaggerated Defensive Reflexes.* The " flinch reflex " and the " jump reflex " which arise from the instinctive actions of defense and flight, have sometimes been diagnosed as hyperacusis. Hyperacusis sometimes occurs with hearing about sixteen times above normal accuity. 100 grains of bromide a day produced no effect on either affliction. Hypnotic suggestion may remove the actual dreams of terror, and yet the reflexes continue unabated. *Treatment.* Complete rest, no visitors, no change of nurses. Confinement to bed and isolation are highly desirable. Hypnotic treatment for [nightmares and day-dreams. Although bromides have little effect, medinal and aspirin produce sleep. Hyperacusis calls for wool plugs and thick pads over the ears, especially when there is much noise or thunder.

III. *Shell Shock.* " The term 'shell shock' should be reserved for the condition which follows exposure to the forces generated by the explosion of powerful shells in the absence of any visible injury to the head or spine. In all cases there is an organic basis, which consists of the more or less evanescent changes in the central nervous system resulting from the concussion caused by aerial compression, to which is often added concussion of the head or spine caused by the sandbags or a falling parapet, or by the patient being blown into the air and falling heavily on his head or back. On this organic basis hysterical and psychasthenic symptoms are often superposed."

*Post-mortem* examination in men who have been blown into the air and have died without regaining consciousness and without apparent injury, discloses punctate hemorrhages in the white matter of the brain, and, in the nerve cells, chromatolysis with eccentric nuclei. Carbon monoxide poisoning from the gas produced by the explosion, gives rise to identical effects, and may be responsible for at least some of the cases, especially when the victims have been confined after explosion in recesses from which the gas did not quickly escape. Although at the base hospitals the cerebrospinal fluid has generally been found normal, French doctors have found by lumbar puncture that, immediately after the explosion, this fluid is under increased pressure, and that it contains albumin, blood, and slight excess of lymphocytes. Within forty-eight hours these symptoms disappear. It is then clear that organic changes occur in the nerves, but they are slight and quickly disappear.

*Cases due entirely to the concussion of the brain and spinal cord.* Shell shock symptoms are identical to those of concussion in civil life. Unconsciousness, stertorous breathing, and, in serious cases, death without recovery of consciousness, are characteristic. Stupor with loss of memory for certain periods occurs in some cases. Nightmare is frequent and headache constant. If there is a raised pressure temporary relief may sometimes result from lumbar puncture. Excitability and irritability often continue long after recovery. Seminal emissions in one case attended the frequent dreams of horror, but were stopped by a single use of hypnotic suggestion.

*Spinal concussion* generally results from burial under earth or sandbags, or sometimes from a blow on the back. A projectile passing near the spine is sometimes the cause of concussion. Roselle and Oberthur have noted that within a few minutes of the injury tendon reflexes were exaggerated and cutaneous reflexes were absent, except for the plantar reflex which was extensor. There was hypotonus of all muscles. In most cases complete recovery with the disappearance of all abnormal signs occurs, but in some cases slight spasticity with exaggerated jerks and occasionally extensor reflexes persists, due to permanent lesion of the spinal cord. Usually there is rigidity and pain upon bending. At first there is often difficulty in the retention of faeces and urine, but later excessive retention. These difficulties soon pass.

*Cases with hysterical symptoms grafted on to organic basis of cerebral or spinal concussion.* Organic paraplegia which is the result of actual spinal injuries, and which would naturally disappear as soon as those injuries have been repaired, is perpertuated in the form of hysterical paraplegia. Suggestion and persuasion may effect immediate cure. When the cure of spinal defects is only partial, spasticity and inaccuracy of movement must to some extent persist. As soon as the initial stupor has passed away an attempt should be made to induce the patient to walk. It is difficult to judge whether or not symptoms are of organic origin. Some of the most marked of these, such as extensor plantar reflex, altered knee jerks, and ankle clonus have proved to be hysterical and therefore quickly amenable to persuasion and reeducation. Autosuggestion is apparently the cause of a good many affections which seem to be the result of lesions of the central nervous system. Men often become blind, deaf, dumb, or hemiplegic, when there it no apparent injury of the nerves. The author explains these divers effects by a theory of hysteria. Since in such cases at first all the faculties are inhibited, the patient upon regaining consciousness notices some one defect, but fails to notice others. By autosuggestion, therefore, one defect, like blindness, may be perpetuated, while the others disappear automatically without having been noticed by the patient at all. It is, therefore, hysterical in nature. However, impressions during the moment between the explosion and loss of consciousness, or those made at the moment of recovery from unconsciousness, may in a few cases explain such phenomena. *Treatment.* Rest is all important *from the start.* Sufficient rest at first often obviates the chronic aches of head and back that follow concussion of the brain or spine, which the most costly treatment administered later fails to cure. Care should be exercised, however, that prolonged rest does not result in hysterical astasia-abasia or paraplegia. Except in severe cases of stupor, the patient should be made to go to the lavatory and to bathe from the first day. When there is no longer pain in the head and back, exercise should be taken, but massage often aggravates the pain and causes introspection.

